# Ionizing Radiation Induces Morphological Changes and Immunological Modulation of Jurkat Cells

**DOI:** 10.3389/fimmu.2018.00922

**Published:** 2018-04-30

**Authors:** Patrick Voos, Sebastian Fuck, Fabian Weipert, Laura Babel, Dominique Tandl, Tobias Meckel, Stephanie Hehlgans, Claudia Fournier, Anna Moroni, Franz Rödel, Gerhard Thiel

**Affiliations:** ^1^Department of Biology, Membrane Biophysics, Technische Universität Darmstadt, Darmstadt, Germany; ^2^Department of Radiotherapy and Oncology, Goethe-University, Frankfurt am Main, Germany; ^3^Department of Biophysics, GSI Helmholtzzentrum für Schwerionenforschung, Darmstadt, Germany; ^4^Department of Biosciences and CNR IBF-Mi, Università degli Studi di Milano, Milano, Italy

**Keywords:** Jurkat cells, peripheral blood lymphocytes, x-ray triggered immune stimulation, T-cell adhesion, x-ray stimulated integrin-β clustering, radiation-induced increase in cell size

## Abstract

Impairment or stimulation of the immune system by ionizing radiation (IR) impacts on immune surveillance of tumor cells and non-malignant cells and can either foster therapy response or side effects/toxicities of radiation therapy. For a better understanding of the mechanisms by which IR modulates T-cell activation and alters functional properties of these immune cells, we exposed human immortalized Jurkat cells and peripheral blood lymphocytes (PBL) to X-ray doses between 0.1 and 5 Gy. This resulted in cellular responses, which are typically observed also in naïve T-lymphocytes in response of T-cell receptor immune stimulation or mitogens. These responses include oscillations of cytosolic Ca^2+^, an upregulation of CD25 surface expression, interleukin-2 and interferon-γ synthesis, elevated expression of Ca^2+^ sensitive K^+^ channels and an increase in cell diameter. The latter was sensitive to inhibition by the immunosuppressant cyclosporine A, Ca^2+^ buffer BAPTA-AM, and the CDK1-inhibitor RO3306, indicating the involvement of Ca^2+^-dependent immune activation and radiation-induced cell cycle arrest. Furthermore, on a functional level, Jurkat and PBL cell adhesion to endothelial cells was increased upon radiation exposure and was highly dependent on an upregulation of integrin beta-1 expression and clustering. In conclusion, we here report that IR impacts on immune activation and functional properties of T-lymphocytes that may have implications in both toxic effects and treatment response to combined radiation and immune therapy in cancer patients.

## Introduction

Ionizing irradiation of eukaryotic cells elicits, in addition to DNA damage and damage responses, also non-targeted effects, which are mainly related to immune activation and immune functional properties (1, 2). An impairment or modulation of the latter has an impact on immune surveillance in both tumor cells and non-malignant cells. This fosters therapy response and unintentional side effects/toxicities as well as an induction of secondary malignancies by radiation therapy (RT) (3, 4). Among the immune cell (sub)populations involved, infiltration of T-lymphocytes, especially cytotoxic CD8+ cells, emerge as valuable prognostic marker for treatment response following RT or multimodal chemoradiation therapy (5, 6) in line with a pro-inflammatory scenario (7, 8). By contrast, a hampered adhesion of peripheral blood lymphocytes (PBL) to the endothelium comprises a major mechanism of the anti-inflammatory effect of low-dose (<1 Gy) RT used in the clinical management of inflammatory and degenerative benign disorders for decades (9, 10).

We have recently reported that an increase of reactive oxygen species (ROS) following X-irradiation of A549 cancer and human embryonic kidney HEK293 cells with doses ≥1 Gy is not restricted to the nucleus but spreads throughout the cell including the cytosol (11). The increase in cytosolic ROS further triggers a Ca^2+^-mediated signal transduction cascade and subsequent activation of Ca^2+^-sensitive channels and membrane hyperpolarization (11, 12). Since a rise in ROS and a downstream triggering of Ca^2+^ signaling cascades may comprise a more general cell response to ionizing irradiation we hypothesize that comparable signaling cascades can be triggered in other types of cells, including immune cells. In line with that it is well established that Ca^2+^ signaling cascades play a crucial role in T-cell activation (13–16) and mediate downstream events like gene expression, entry into the cell cycle and T-cell effector functions. Notably, these signaling cascades can be short-circuited by elevating the concentration of free Ca^2+^ in the cytosol ([Ca^2+^]_cyt_) without employing receptor activation (17).

With this background information, we analyze here the effect of ionizing radiation (IR) with low (<2 Gy) and higher doses (≥2 Gy) on morphological changes, immune activation, adhesion properties, and ion channel expression of a leukemic Jurkat T-cell line and PBL. The Jurkat cell line has served for two decades as a valuable model for analyzing basic signaling events engaged in T-cell activation (17). Our data indicate that irradiation of Jurkat and PBL cells triggers a series of distinct cellular responses. These include an increase in cell diameter, augmented integrin β1-mediated adhesion to endothelial cells (ECs), CD25, interferon-γ (IFNγ), and interleukin (IL)-2 stimulation and modulation of Ca^2+^ sensitive K^+^ channels.

## Materials and Methods

### Cell Culture

Jurkat cells (ACC 282) were purchased from the German Collection of Microorganisms and Cell Cultures (DSMZ, Braunschweig, Germany). The human EC line EA.hy926 (Crl-2922) was established by fusion of human umbilical vein ECs and the adenocarcinoma epithelial cell line A549 (18) and was purchased from ATCC (LGC Standards, Wesel, Germany). Cells were either grown in RPMI 1640 medium (Jurkat), supplemented with 10% heat inactivated fetal calf serum (FCS; PAA, Cölbe, Germany) and 2 mM l-glutamine or in Dulbecco’s modified Eagle’s medium (Invitrogen, Karlsruhe, Germany), supplemented with 10% FCS 50 U/ml penicillin and 5 µg/ml streptomycin (Sigma-Aldrich, Munich, Germany). PBL were isolated from buffy coats using density gradient centrifugation (Biochrom, Berlin, Germany). After centrifugation (40 min at 1,000 × *g*, RT) interphase cells were isolated, washed twice with PBS, and pelleted by centrifugation (300 × *g*, 10 min). For adhesion assays, Jurkat cells and PBL were biotinylated by incubation (15 min on ice) with a biotin-*N*-hydroxysuccinimid ester (NHS-biotin, 10 mg/ml, Sigma-Aldrich) and maintained in RPMI 1640 Medium with 20% FCS, 1% HEPES, and 1% penicillin/streptomycin prior to assays. PBL isolation was performed in a biolevel II laboratory with an institutional approval by the local governmental authority (Regierungspräsidium Darmstadt IV/F-45.1/jr-F 018164-23623/2017-Bio-30/17).

### Determination of Cell Diameters

Cell diameters were measured with an EVE automatic cell counter (NanoEnTek, Seoul, South Korea). For cell diameter studies, a suitable protocol for Jurkat cells was established and all measurements were validated by visual inspection and if necessary corrected by hand using a personal computer based software. Viability was estimated by using trypan blue exclusion assays.

### Cell Irradiation and Treatments

Cells were exposed to X-ray irradiation in cell culture flasks using an Isovolt 160 Titan E source with a voltage of 90 kV and 33.7 mA (GE Sensing & Inspection Technologies, Alzenau, Germany). Doses were delivered at a 30 cm source to probe distance with cell culture flasks placed on a 2 mm aluminum sheet. CDK1-inhibitor RO3306 (Axon Medchem, Groningen, Netherlands) was dissolved in DMSO at 14.2 mM and added to the cell culture medium in a final concentration of 3 µM. Cyclosporin A (Sigma-Aldrich) was dissolved in ddH_2_O and added to the cell culture medium of non-irradiated control cells or directly after irradiation of cells in a concentration of 1 µM. The cell permeable Ca^2+^ buffer BAPTA-AM [*1,2-Bis(2-aminophenoxy)ethane-N,N,N*'*,N*'*-tetraacetic acid tetrakis(acetoxymethyl) este*r, Thermo Fisher] was added to the cell culture medium 30 min prior to cell irradiation at 50 µM and was removed immediately after irradiation. Phytohemagglutinin (PHA-L) was purchased from Biochrom (Berlin, Germany). Cells were treated for 48 h by adding PHA-L to the cell culture medium at a concentration of 7.2 µg/ml. To activate human T-cells ImmunoCult™ Human CD3/CD28/CD2 T Cell Activator (Stem cell Technologies, Vancouver, BC, Canada) was added to the cell culture medium (25 µL per 1 mL of cell suspension) and maintained at 37°C and 5% CO_2_ for 48 h. The K_Ca_2.2-specific ion channel blocker Tamapin was purchased from Alomone Labs (Jerusalem, Israel) and dissolved in purified water and diluted in external solution for patch clamp experiments.

### Immunofluorescence

#### Staining of IFNγ and IL-2 for Immune-Fluorescence Detection

4 × 10^5^ Jurkat cells/ml were treated with either 25 µl/ml CD3/CD28/CD2 T-cell activator or irradiated with X-ray doses between 0.1 and 5 Gy. After 48 h incubation at 37°C, 5% CO_2_ the cell suspensions were washed with PBS at 400 × *g* for 5 min. Next, the cells were fixed for 30 min at room temperature in 4% paraformaldehyde (PFA) with 0.2% glutaraldehyde in PBS and permeabilized with 0.2% Triton X-100 solution. T-cell suspensions were washed in PBS, resuspended in PBS and primary antibodies for IFNγ (#14-7317-85, Thermo Fisher Scientific, Waltham, MA, USA) or IL-2 (#92381, Abcam, Cambridge, UK) were applied at a 1:2,500 dilution over night at 4°C on a shaker. Jurkat cells were subsequently washed with 0.05% Tween20 (in PBS) and incubated with anti-mouse Alexa488 secondary antibody (anti-mouse Alexa488 IgG, Thermo Fisher Scientific) in a dilution of 1:2,500 for 1 h at RT. Finally, stained cells were washed with 0.05% Tween20 (in PBS) and stored in PBS before analysis. For an analysis of IL-2 and IFNγ expression by immunostaining untreated control cells and cells irradiated with X-ray or treated with activator were imaged with the same microscope settings. For a quantitative analysis, a region of interest (ROI) was defined and fluorescence intensity was measured relative to the size of the ROI.

### Integrin β1 and K_Ca_2.2 Staining for Single Molecule Analysis

Cell fixation and antibody staining were performed as described earlier (19). In brief, Jurkat cells were fixed with a rapid and complete immobilization fixation protocol optimized for membrane proteins (20). Cells were incubated in 4% PFA supplemented with 0.2% glutaraldehyde for 1 h at 4°C followed by anti-integrin β1 (CD 29, Biozol Diagnostica, Eching, Germany) immunostaining with a directly fluorescent labeled antibody (Alexa 488). K_Ca_2.2 channels were stained with KCNN2 antibody (PA5-41012, rabbit IgG, Thermo Fisher Scientific) as primary antibody and with an Alexa 488 labeled anti rabbit secondary antibody (Thermo Fisher). In both procedures an antibody dilution of 1:10,000 was used.

### Western Immunoblotting

For Western blotting, cells were lysed in radio-immune precipitation assay buffer supplemented with protease inhibitors. Equal amounts of proteins (30 µg) as determined by a micro BCA-protein assay (Pierce, Rockford, IL, USA) were separated on 12% SDS polyacrylamide gels and transferred to a nitrocellulose membrane (Hybond C, Amersham, Freiburg, Germany). Membranes were next incubated with rabbit anti-CD25 antibodies (S-IL2R Oligo, Life Technologies, Darmstadt, Germany). This was, followed by an incubation with appropriate horseradish peroxidase-conjugated secondary antibodies (Southern Biotech, Birmingham, AL, USA). Next, membranes were developed by using an enhanced chemo luminescence detection system (ECL, Perkin Elmer, Waltham, MA, USA) and Odyssey Fc Imaging System (LI-COR, Bad Homburg, Germany). To confirm equal protein loading, membranes were in parallel probed with anti β-actin antibodies (Sigma-Aldrich). Individual bands were quantified using the Image Studio Version 5.2 (LI-COR).

### Confocal Laser Scanning Microscopy

Confocal laser scanning microscopy was performed on a Leica TCS SP or SP5 II system (Leica Microsystems, Mannheim, Germany) equipped with a 63× water (HCX PL APO 63× NA 1.2 W CORR) and 63 × 1.4 oil UV objective (HCX PL APO lambda blue). Coverslips were cleaned using acetone followed by plasma cleaning in a plasma furnace (Zepto-B) from Diener electronic (Ebhausen, Germany). The external buffer used for microscopy contained (140 mM NaCl, 4 mM KCl, 1 mM MgCl_2_, 5 mM Mannitol, 10 mM HEPES, 2 mM CaCl_2_, pH 7.4). Plasma membranes were imaged with CellMaskOrange™ (Thermo Fisher Scientific) at a concentration of 0.5 µg/ml. Nuclei were stained with Hoechst (200 µg/ml) diluted 1:50 in external microscopy buffer or PBS; cells were stained for 10 min at 37°C. Subsequently, cells were washed twice and resuspended in microscopy buffer or PBS.

### Ca^2+^ Imaging

The sensor Fluo-4 was loaded into Jurakt cells by incubating cells for 30 min in buffer (140 mM NaCl, 4 mM KCl, 1 mM MgCl_2_, 5 mM Mannitol, 10 mM HEPES, 2 mM CaCl_2_, pH 7.3) containing 1 µM Fluo-4 AM (Life technologies, Carlsbad, CA, USA) on coated glass coverslips (Ø 25 mm). The latter were prepared by cleaning in a plasma furnace (Zepto-B, Diener electronic GmbH, Ebhausen, Germany) and coating with one layer of PBS/5% BSA in a spincoater (PIN150, SPS Europe Spincoating, Putten, Netherlands). After the initial layer had dried, it was further coated with a layer of poly-L-lysine (molecular weight 75–150 kDa). Coating was essential to prevent spontaneous Ca^2+^ oscillations, which usually occur when Jurkat cells are settling on glass coverslips. The dye was subsequently removed by washing cells with dye free buffer. After irradiation, the cells were then transferred for imaging on a Leica TCS SP5 II confocal microscope (Leica, Heidelberg, Germany) with a HCX PL APO CS 40.0 × 1.30 OIL oil immersion lens. The dye was excited with a 488 nm argon laser and the emission sampled at 505–550 nm.

### Single Molecule Microscopy and Data Analysis (SMD)

For SMD measurements a standard STORM buffer containing 100 mM MEA (β-mercapto ethylamine, pH 8.5, Sigma-Aldrich, St. Louis, MO, USA), 140 U catalase (Sigma-Aldrich, St. Louis, MO, USA, C3515), and 10 U glucose oxidase (Sigma-Aldrich, St. Louis, MO, USA, G0543) in Tris-buffer [50 mM Tris, 10 mM NaCl (both AppliChem, Darmstadt, Germany), pH 8] supplemented with 10% (w/v) glucose was used. All SMD measurements were performed with a custom built instrument. A detailed description of this setup and the data analysis of detected molecules were published elsewhere (19). In brief, editing of images was performed with Fiji software (version: 1.51h) (21). Single molecules were detected and filtered using the Thunder Storm plugin for Fiji (22). For the add-on data analysis, custom written software in MATLAB R2014b was used. Therefore, Ripley’s K function cluster analysis (23, 24) was combined with a binary cluster map analysis based on the publication of Owen et al. (25). With this add-on it is possible to (i) determine the number of molecules per ROI and (ii) the ratio of clustered/total signals. Detected single molecules are visualized as Gaussian rendered images. Here, a symmetric 2D Gaussian is drawn for every localized molecule with a SD equal to the localization uncertainty. The localized and rendered molecules are added sequentially leading to the final super-resolution image. To remove duplicates, molecules that convert to the positions within a distance of the uncertainty were removed.

### Patch Clamp Recordings

Membrane currents of cells were recorded in a whole cell configuration (26) using an EPC-9 amplifier (HEKA Electronics, Lambrecht, Germany). The pipette solution contained (in mM) 100 K-Aspartate, 40 KF, 5 KCl, 2 MgCl_2_, 1.223 EGTA (1 µM free Ca^2+^) or 2.62 EGTA (100 nM free Ca^2+^), 1 CaCl_2_, and 10 Hepes/KOH pH 7.4. Sorbitol was used to adjust the osmolarity to 285 mOsmol/kg. The extracellular solution contained (in mM) 130 Na-Asp, 4.5 KCl, 1 MgCl_2_, 2 CaCl_2_, and 10 Hepes/NaOH pH 7.4. Currents were elicited with a pulse protocol consisting of voltage steps from a holding voltage at −60 mV, to 800 ms long test pulses between −100 and +80 mV and a 200 ms long post pulse at −80 mV. Currents were recorded and data analyzed with an EPC-9 amplifier and Patchmaster Software (all from Heka Electronic).

### Cell Cycle Analysis by Flow Cytometry

Flow cytometric analyses were performed after 48 h following irradiation with propidium iodide (PI) solution (4% PI stock, 0.5 mg/ml PI, 38 mM sodium citrate, pH 7), 5% RNAse A stock (RNAse A 5 mg/ml, Tris–HCl 10 mM, NaCl 15 mM, pH 7) using a BioRad S3 Cell Sorter and the FlowJo 10 software for analysis (FlowJo LLC). The percentage of cells in G_2_/M phases was determined by single-parameter histograms of DNA content.

### Cell Adhesion Assay

EA.hy926 EC were grown to 95% confluence and stimulated by the cytokine TNF-α (20 ng/ml, MiltenyiBiotec, Bergisch-Gladbach, Germany) at 4 h before the adhesion assay. Next, a total of 2–3 × 10^5^ irradiated and biotinylated Jurkat cells or PBL were added and adhesion assays were performed for 30 min at 4 or 37°C under non-laminar shear stress as reported before (27). Next, adherent PBL or Jurkat cells were fixed with methanol, tagged with a streptavidin-Cy3 conjugate (Dianova, Hamburg, Germany) and counted using an Operetta High Content Screener (PerkinElmer, Waltham, MA, USA). The counts of a minimum of 160 selected fields per well were averaged as one data point.

### CD25 Detection by Flow Cytometry

Surface expression of CD3 and CD25 was analyzed either on Jurkat cells harvested directly from cultures flasks or PBL isolated by density gradient centrifugation as described before. Next, cells were stained with fluorochrome-conjugated mAb targeting CD3 (CD3-PerCP-Cy5.5 clone SK7; Becton Dickinson, Heidelberg, Germany) and CD25 (BV510 Mouse anti human CD25 clone 2A3, Becton Dickinson) and subjected to multicolor flow cytometry using a CytoFlexS cytometer (Beckman Coulter, Krefeld, Germany). Data acquisition and analysis were accomplished with CytExpert Version 1.2 software (Beckman Coulter).

### Taqman-Based Quantitative Real-Time PCR (qRT-PCR)

RNA was isolated at 24 h post irradiation or T-cell activation using the NucleoSpin Kit (Macherey-Nagel, Dueren, Germany) in combination with the QiaShredder Kit (Qiagen, Hilden, Germany) according to the manufacturer’s recommendations. Reverse transcription was performed with M-MLV reverse transcriptase (Promega, Mannheim, Germany) and random hexamers (Thermo Fisher Scientific). qRT-PCR was achieved with 20× Taqman Assays (Thermo Fisher Scientific) specific for IL-2 (Assay ID: Hs00174114_m1) or IFNγ (Assay ID: Hs00989291_m1) with the StepOnePlus Real-Time PCR System (Thermo Fisher Scientific), ABsolute QPCR Mix, ROX (Thermo Fisher Scientific) and standard settings. Relative gene expression was calculated using the 2^−ΔΔCt^ method relative to untreated controls with the housekeeping gene ribosomal protein L37A (RPL37A) as endogenous reference. For each data point, two independent experiments performed in triplicate were acquired and displayed as mean value + SD. The primer and probe sequences for RPL37A detection were as follows: RPL37A-fw 5′-TGTGGTTCCTGCATGAAGACA-3′, RPL37A-rev 5′-GTGACAGCGGAAGTGGTATTGTAC-3′, RPL37A probe: 5′ FAM-TGGCTGGCG GTG CCT. GGA-3′ TAMRA (28), manufactured by Eurofins Genomics (Ebersberg, Germany).

### Statistical Analysis

Data are expressed as means ± SDs or SE of at least two independent experiments; number of biological replicates (*n*) or independent experiments (*N*) were denoted. Significance was estimated by using the Student’s *t*-test and Microsoft Excel software. *P* values <0.05 (*), <0.01 (**), and <0.001 (***) are indicated in the figures.

## Results

### Ionizing Irradiation Increases Cell Diameter of Jurkat Cells and PBL

**Figure 1:**
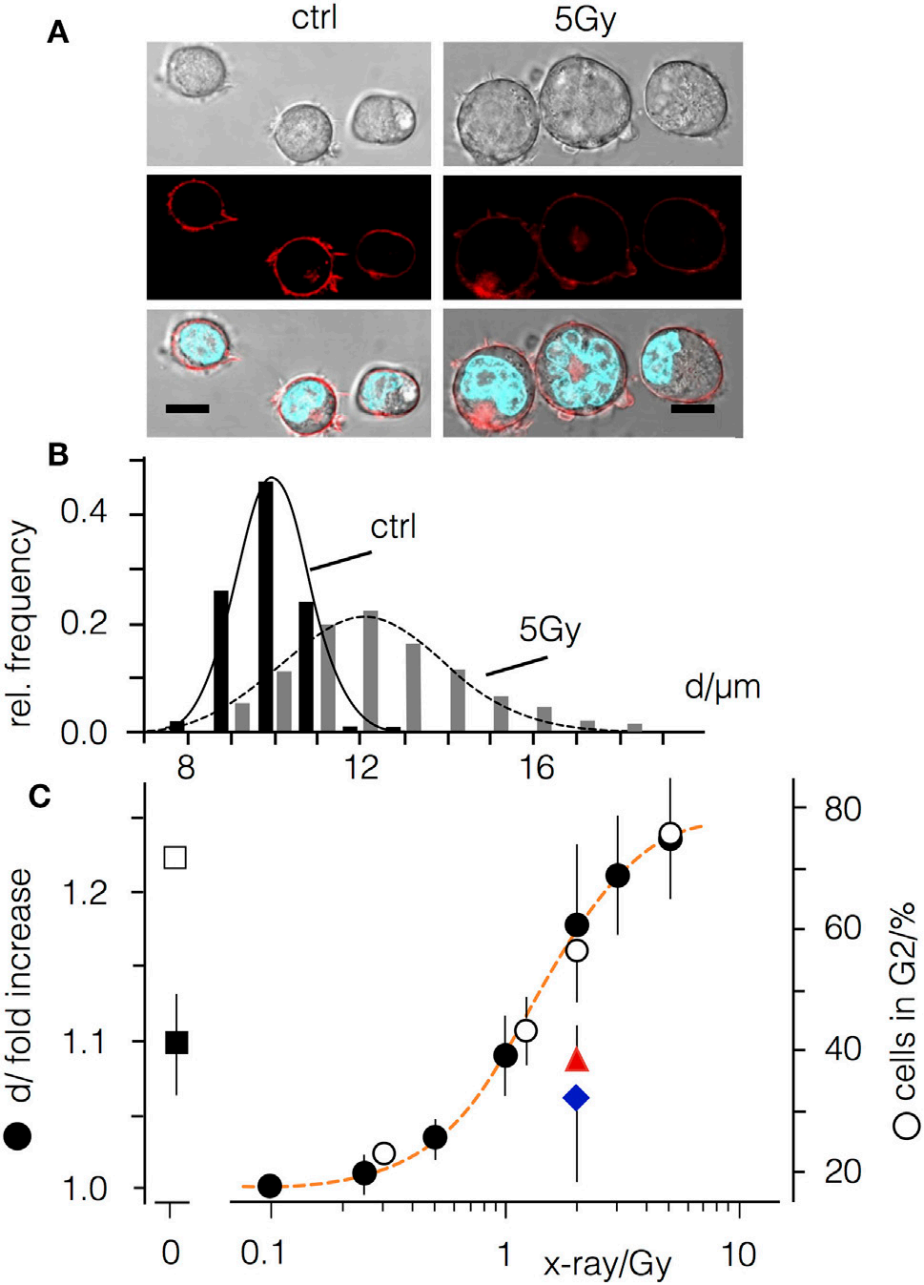
Ionizing irradiation causes an increase in diameter of Jurkat cells and an arrest in G2. **(A)** Images of Jurkat cells before and 48 h after X-irradiation with 5 Gy. Top row: wide field image, central row: confocal section in equatorial plane with fluorescent plasma membrane label CellMaskOrange™. Lower row: overlay of wide field image with CellMaskOrange™ labeled plasma membrane and Hoechst labeled nucleus; scale bars 10 µm. **(B)** Cell diameter distribution of non-irradiated Jurkat cells (black bars) and cells irradiated with 5 Gy (gray bars). **(C)** Relative increase of cells in G2 phase of the cell cycle (open circles) and increase in cell diameter (d, closed symbols) in the absence (black circles) or presence of either 1 µM cyclosporine A (red triangle) or 50 µM BAPTA-AM (blue diamond). Square symbols show relative increase in cell diameter (closed symbol) and percentages of cells in G2 in non-irradiated cells treated with 3 µM CDK1-Inhibitor RO3306 (open symbols).

Jurkat cells exhibit a narrow size distribution with a mean value of 10.1 ± 0.2 µm (Figures 1A,B). Forty-eight hours after a 5 Gy exposure the distribution widens and the mean value increases to 12.5 ± 0.5 µm. Comparable findings were also evident following irradiation with doses ranging between 0.1 and 10 Gy (Figure 1C) confirming a dose-dependent increase in the mean cell diameter (Δd). A fit of the plot with a logistic equation (Eq. 1)

(1) $$f(x)=\frac{\Delta d_{\text{max}}}{{\left(\frac{D50}{x}\right)}^{k}+1}$$

where Δ*d*_max_ is the maximal Δ*d* increase, *D*50 the dose for half maximal increase and *k* the steepness of the curve, yields a *D*50 value of 1.34 Gy with a steepness of 2; the curve saturates at a maximal Δ*d* of 23.5% for doses ≥5 Gy.

**Figure 2:**
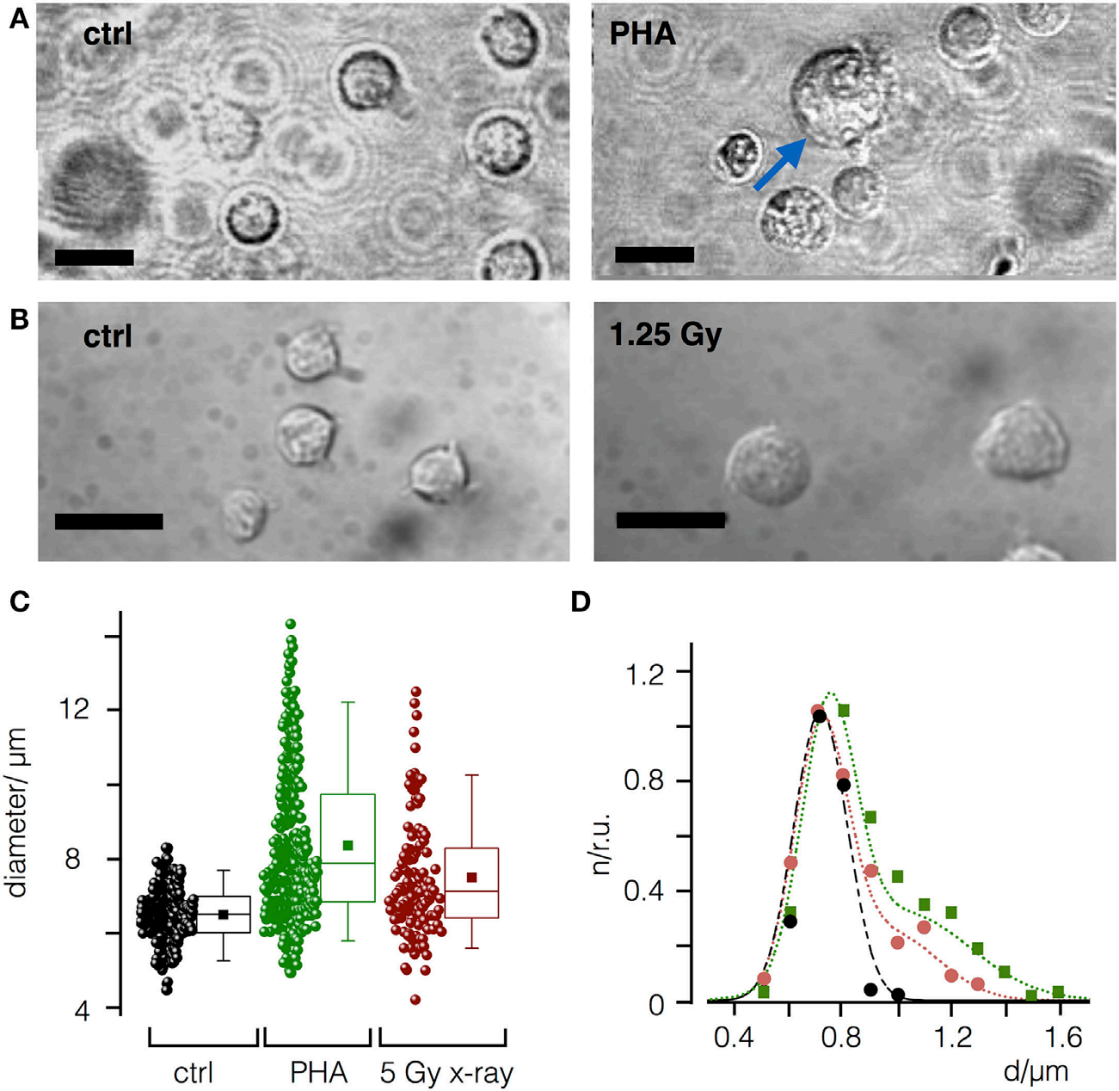
Mitogen phytohemagglutinin (PHA-L) and ionizing irradiation cause an increase in diameter of naïve peripheral blood leukcocytes (PBL). **(A)** Representative images of PBL before (control) and 48 h after treatment with PHA-L (7.2 µg/m). **(B)** Before and 48 h after irradiation with a dose of 5 Gy. Scale bars 10 µm. **(C)** Cell diameters of PBL control cells (ctrl) or PBL treated with PHA-L or 5 Gy. Each data point represents a single PBL with mean (filled square) and median value (line) as well as 25 and 75 percentile of data; whiskers indicate 5 and 95 limits of data. **(D)** Size distribution histogram of ctrl (black) and of cells treated with PHA-L (green) or 5 Gy (red) from **(B)**. Distribution was normalized to maximal value for each condition and fitted with single Gauss distribution (control, black line) or the sum of two Gaussians for T-cell activator (green line) or X-ray (red line) treated cells.

It has been shown that peripheral blood leukocytes increase in size in response to PHA-L immune stimulation (29). Accordingly, we next asked whether IR may increase cell diameter in a comparable manner. Indeed, as depicted in Figures 2A–C stimulation of PBL from healthy donors (*N* = 3) with PHA-L (30) resulted in a comparable increase in cell diameter. The size distribution of mock-treated and irradiated cells can be fitted by either a single Gaussian distribution confirming a uniform size with a mean diameter of 7.2 µm (mock treated) or by the sum of two Gaussian distributions. The two populations indicate that following PHA-L stimulation 45% of the cells have increased their mean size to 10.2 µm, while 48 h after irradiation with a dose of 5 Gy 32% of the cells exhibit an increased diameter (mean value at 9.7 µm).

### Effect of Ionizing Irradiation on Cell Cycle Distribution in Jurkat Cells

Jurkat cells are deficient in p53 (31) and consequently an irradiation-induced arrest is restricted to the G2 phase of the cell cycle (Figure 1C), which is associated with an increase in the size of the cell nucleus (Figure 1A). The distribution of cells in G2 phase exhibits a similar dose-dependency as the increase in cell size (Figure 1C). To test whether these two parameters are related, Jurkat cells were treated with the CDK1-inhibitor RO3306. Incubation with RO3306 arrested 71% (±2.1%) of the cells in G2 phase (Figure 1C) but only resulted in a 9.6 ± 3% increase in cell diameter (Figure 1C). Irradiation with a dose of 5 Gy, by contrast, revealed a similar accumulation in the G2 phase (76.3 ± 6%) but with an increase in diameter of 24% (±4%) (Figure 1C).

Next, we co-treated irradiated Jurkat cells (2 Gy) with cyclosporine A or the Ca^2+^ buffer BAPTA-AM. We reasoned that immune suppression or blocking the Ca^2+^ signaling cascades may abolish the radiation-induced increase in cell diameter without affecting the G2 cell cycle arrest (Figure 1). Indeed, both treatments decreased the effect of irradiation (Figure 1C) with an increase of diameter in cyclosporine A treated cells of 10 ± 3%, as compared to 19 ± 5% in mock-treated controls. Notably the remaining value of 8% increase was comparable to the value induced by the CDK1-inhibitor (Figure 1C).

**Figure 3:**
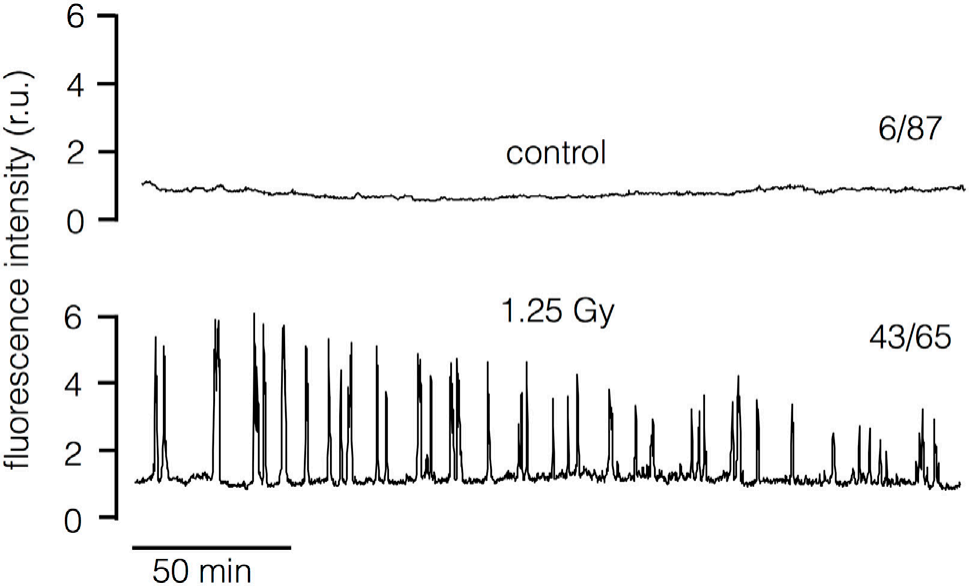
Ionizing irradiation stimulates oscillations of cytosolic Ca^2+^ in Jurkat cells. Representative recordings of Fluo-4 fluorescence in untreated control cell (top) and in cell after exposure to 1.25 Gy x-Ray (bottom). The recordings start approximately 7 min after exposure to radiation. The numbers of cells (*n*) which show [Ca^2+^]_cyt_ oscillations 1 h after irradiation (1.25 Gy) or after sham treatment (control) with respect to total number of cells investigated (*N*) is reported at respective traces (*n*/*N*). Data are pooled from three independent experiments.

The sensitivity of the irradiation triggered morphological response of Jurkat cells to the Ca^2+^ buffer BAPTA-AM suggests that a Ca^2+^-mediated signaling cascade is connecting the primary radiation stress and the morphological alteration. To test this prediction we loaded Jurkat cells with the Ca^2+^ sensitive dye Fluo-4 and imaged the concentration of free Ca^2+^ in the cytosol [Ca^2+^]_cyt_ in untreated cells and with 1.25 Gy irradiated cells. The representative recordings of the Fluo-4 fluorescence in Figure 3 indicate that the signal remains constant in the majority of control cells but starts oscillating after a delay of about 30 min in most irradiated cells. 1 h after irradiation with 1.25 Gy, 67% of the treated cells exhibited oscillations in [Ca^2+^]_cyt_. In the respective control cells, only 7% exhibited an oscillation at this time point of recording. The results of these experiments confirm that ionizing irradiation triggers in Jurkat cells a Ca^2+^ signaling cascade, which is initiated only after a considerable delay.

**Figure 4:**
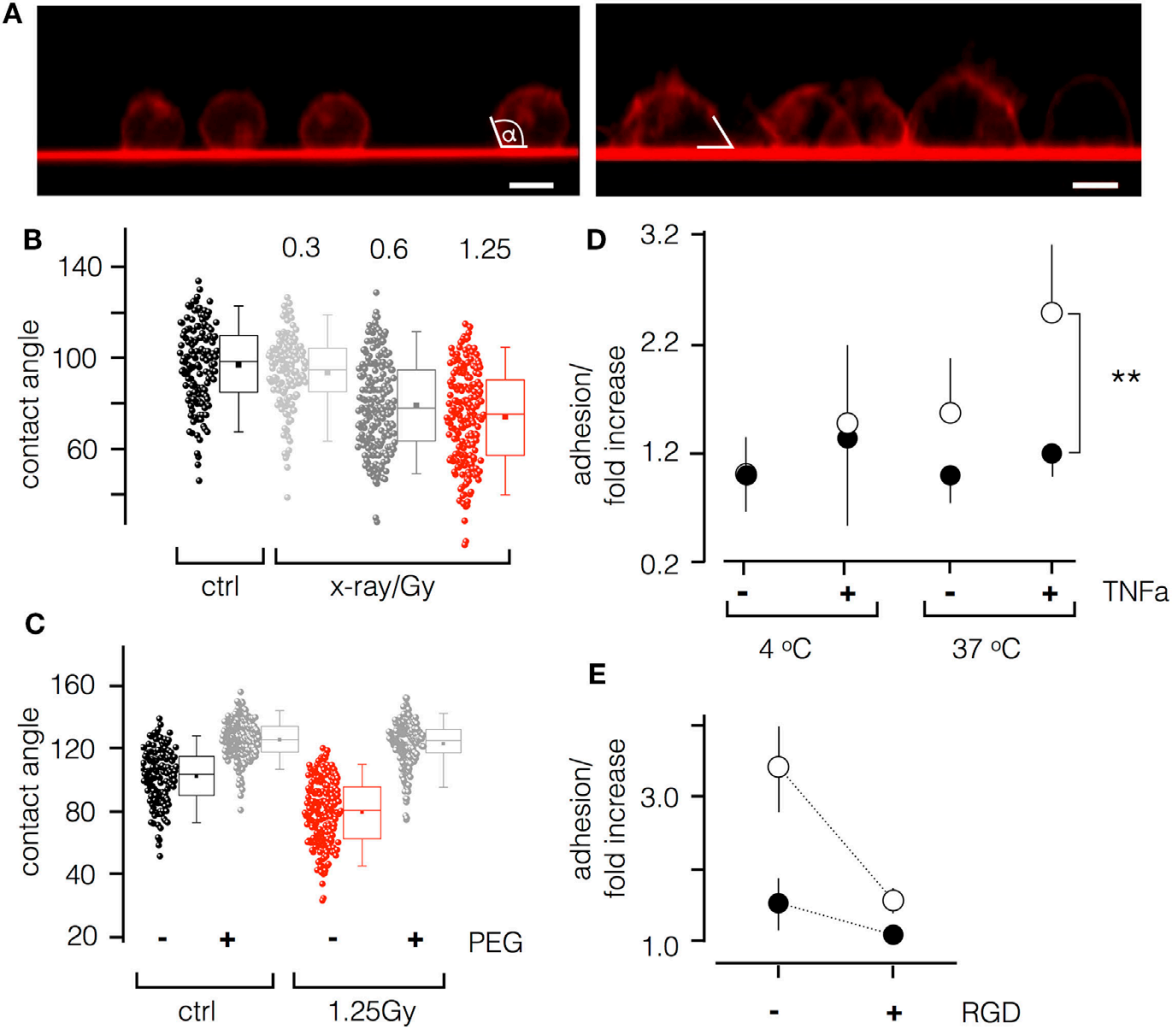
Ionizing irradiation stimulates adhesion of Jurkat cells and peripheral blood lymphocytes (PBL). **(A)** Side view of Jurkat cells with fluorescent plasma membranes on glass surface. Confocal images of non-irradiated cells (left panel) and of cells 48 h after irradiation (1.25 Gy, right panel) were taken 10–15 min after incubating cells on red fluorescent glass cover slip. White lines indicate the contact angle between cell and glass surface. **(B)** Box plot of contact angles for un-irradiated cells and cells irradiated with increasing doses of X-ray. **(C)** Contact angles of un-irradiated (ctrl) and irradiated cells (1.25 Gy) on untreated (−) or polyethylene glycol (PEG) pretreated (+) cover slips. Data obtained as in **(B)**. Box plot symbols have the same meaning as in Figure 2. **(D)** Relative adhesion rates of Jurkat cells and PBL to endothelial cells (ECs). Non-irradiated (closed circles) or irradiated (1.25 Gy X-ray) Jurkat cells at 4 or 37°C with or without stimulation of ECs with TNF-α (20 ng/ml). Mean value ± SD (*N* = 4; *n* = 12). **(E)** Cells as in last column of **(D)** with or without 10 µM RGD peptide in incubation buffer.

To further analyze irradiation-induced morphological changes of Jurkat cells, we imaged them for 48 h after exposure to 1.25 Gy. As depicted in Figure 4A, non-treated cells were spherical with a small foot on the glass surface. This foot area became much larger in irradiated cells. This suggests that the inherent tendency of Jurkat cells to adhere to the glass surface was accelerated by ionizing irradiation. To further quantify adhesion on glass surface, we estimated the contact angle between the cell and the glass (Figure 4A). As shown in Figure 4B, irradiation triggers a significant (*P* < 0.001) decrease in the contact angle from 101.1° ± 17.7° in control cells to 78.3° ± 20.8° in irradiated cells. To test whether this effect is the consequence of cell adhesion, experiments were repeated on polyethylene glycol (PEG)-coated glass coverslips. Figure 4C illustrates that PEG coating increases the contact angle due to a decreased cell-surface adhesion. This tendency is strongly accelerated by X-irradiation of the cells (Figures 4B,C).

Next, to test whether ionizing irradiation also stimulates cell adhesion in a more physiological context, we performed an adhesion assay on EA.hy926 ECs. As depicted in Figure 4D, Jurkat cells or PBL irradiated with a dose of 1.25 Gy exhibited an elevated adhesion rate to EA.hy926 cells, which was most pronounced for both cell types after stimulation of the ECs with the pro-inflammatory cytokine TNF-α. Moreover, to analyze the involvement of integrin adhesion molecules, Jurkat cells or PBLs were incubated with recognition sequences Arg–Gly–Asp (RGD) peptides to compete for binding of endothelial-leukocyte adhesion molecules and vascular cell adhesion molecule receptors. Results presented in Figure 4E indicate a significant reduction of adhesion in the presence of the peptides, indicating a mechanistic impact of RGD motifs.

The same types of adhesion assays were performed with PBL revealing a similar response of these cells to irradiation (Figure S1 in Supplementary Material). 1.25 Gy significantly augments adhesion to ECs in particular in TNF-α stimulated ECs; the response is comparable to that elicited by a T-cell activator. Also in these experiments, the Arg–Gly–Asp peptide caused a reduction of radiation-triggered adhesion suggesting an involvement of integrins.

**Figure 5:**
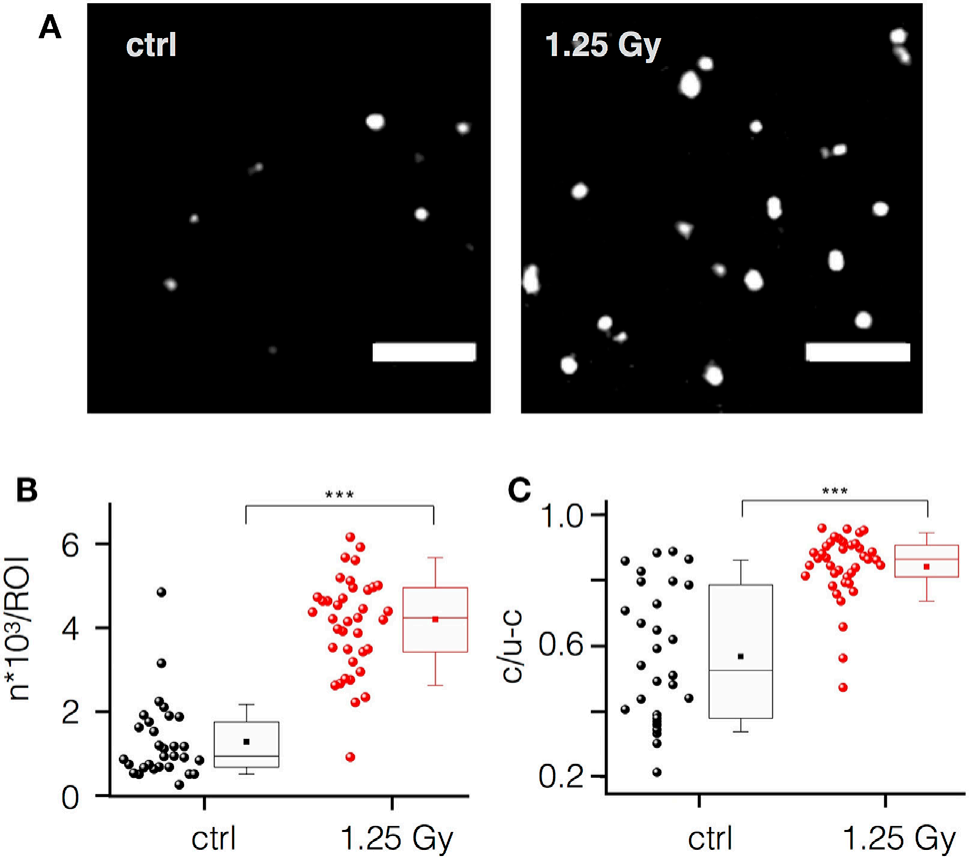
Ionizing irradiation increases integrin β1 expression and clustering in Jurkat cells. **(A)** Localization of single integrin β1 molecules and their organization as clusters in plasma membrane areas of non-irradiated (control) and irradiated (1.25 Gy) Jurkat cells visualized by high-resolution microscopy (scale bar = 1 µm). **(B)** Mean number of molecules per region of interest (ROI) in control cells (black circles) and in irradiated cells (red circles). **(C)** Ratio of clustered versus non-clustered integrin β1 molecules in irradiated and non-irradiated cells. Each point in **(B,C)** represents data from one ROI of an image. Box plot symbols have the same meaning as in Figure 2.

To confirm an integrin-mediated adhesion, we imaged the integrin β1 subunit in non-irradiated and irradiated (1.25 Gy) Jurkat cells with single molecular resolution. Representative images in Figure 5A visualize a significant increase of integrin β1 molecules and cluster detection upon irradiation by quantitative evaluation (Figures 5B,C). Here, single molecules of integrin β1 are visualized as Gaussian rendered images. For this, the localized and rendered molecules are added sequentially resulting in a better visualization of regions with a higher density of signals. These regions, shown as white spots, are well known as integrin clusters (32). Quantitative analysis with the Ripley’s K function supports the visual impression of an irradiation-induced increase in the density of clusters and number of integrin β1 molecules. In addition, size of the clusters is larger in irradiated cells as compared to control cells (Figures 5B,C).

**Figure 6:**
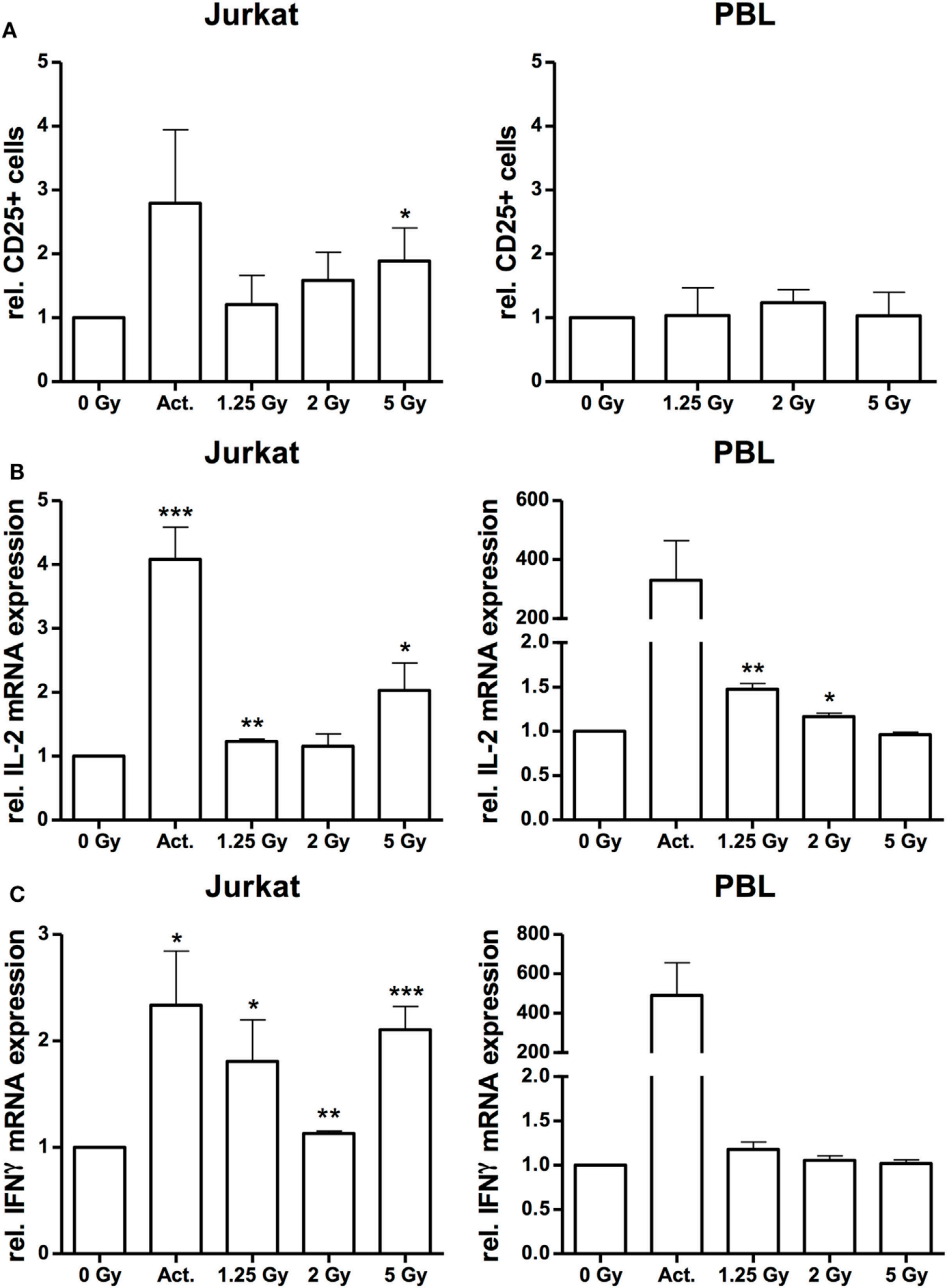
Irradiation stimulates immune activation in Jurkat cells and peripheral blood lymphocytes (PBL). FACS analysis of CD25 surface expression on Jurkat cells and CD3-positive PBL **(A)** following irradiation with a dose of 1.25, 2, and 5 Gy. Stimulation with 25 µl/ml CD3/CD28/CD2 T-cell activator (Act.) in Jurkat cells or mock-irradiated cells served as controls (*N* = 3). In PBLs, an activator could not be applied due to inference with the CD3 stimulus. Quantification of interleukin (IL)-2 **(B)** and interferon-γ (IFNγ) **(C)** mRNA expression by quantitative real-time PCR in Jurkat cells and PBL at 24 h after irradiation with a dose of 1.25, 2, or 5 Gy. Stimulation with 25 µl/ml CD3/CD28/CD2 T-cell activator (Act.) or mock-irradiated cells served as controls (*N* = 2). Data are represented as mean + SD. Student’s *t*-test compared activator-treated or irradiated cells with non-irradiated controls; *P < 0.05, **P < 0.01, ***P < 0.001.

Up to this point, the data so far supported the hypothesis that ionizing irradiation induces morphological changes and increases adhesion of Jurkat cells and PBL, which may resemble immune activation processes (33, 34). To further analyze the effect of X-irradiation on Jurkat cell activation, we monitored the surface expression of CD25 (IL-2 receptor alpha chain), and IL-2 and IFNγ response by FACS analyses and quantitative PCR, respectively. The results of these assays indicate a dose-dependent increase of CD25 expression by X irradiation in Jurkat cells while the number of CD25+ cells in PBL was not affected (Figure 6A). Quantitative analysis of IFNγ and IL-2 mRNA revealed an increased expression in Jurkat cells, most pronounced following a 5 Gy exposure (Figures 6B,C). By contrast, as compared to a huge activation level by the CD3/CD28/CD2 cocktail, we observed a low IL-2 (Figure 6B) or marginal radiation-dependent induction of IFNγ in native PBL (Figure 6C). Increased induction of either CD25, IL-2 or IFNγ in Jurkat cells was further confirmed by Western-Blot analyses and immuno-fluorescent detection and quantification (Figure S2 in Supplementary Material).

Finally, immune activation is reported to upregulate Ca^2+^ sensitive K^+^ channels in immune cells for differentiation and activation (35). In Jurkat cells, the K_Ca_2.2 (SK2) channel is activated by elevated [Ca^2+^]_cyt_ concentration and may serve as a target of an IR-induced Ca^2+^ signaling cascade. To examine the effect of IR on channel expression and activity, we analyzed channel currents 48 h after X-ray exposure where the increase in cell diameter and the expression of CD25 is most pronounced.

**Figure 7:**
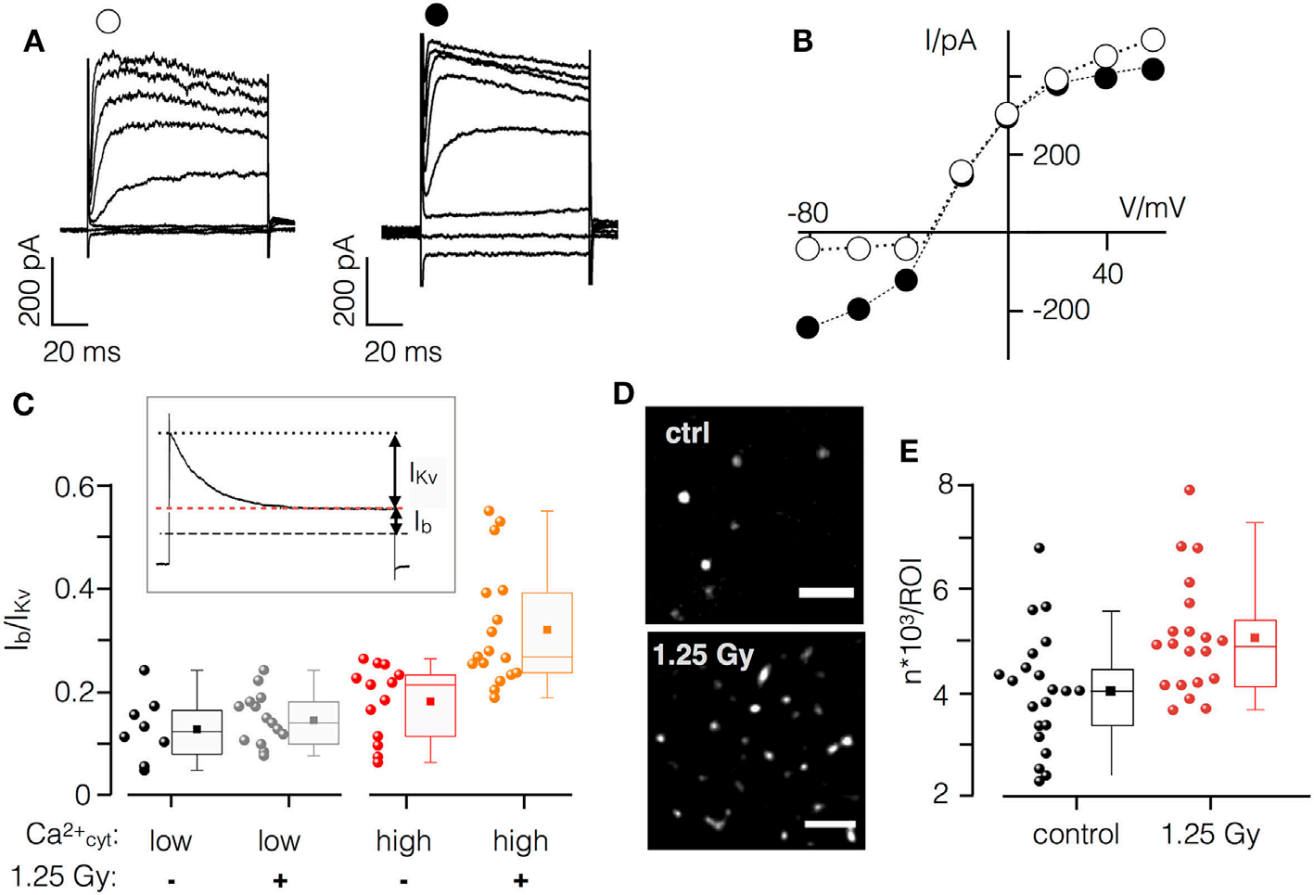
X-ray irradiation activates Ca^2+^ sensitive K^+^ channel in Jurkat cells. **(A)** Current responses of un-irradiated (left) and irradiate (right) Jurkat cell in whole cell configuration with high (1 µM) cytosolic Ca^2+^ to test voltages between −80 and +60 mV. **(B)** Peak current/voltage relation of cells in **(A)**. Symbols in **(A)** correspond to symbols in I/V plot. **(C)** Inset: during long clamp steps, the time-dependent Kv1.3 current inactivates (I_Kv_) leaving the voltage-independent background current I_b_. Ratio of I_b_/I_Kv_ from non-irradiated (−) and irradiated (+) Jurkat cells (1.25 Gy) with low (<100 nM) or high (1 µM) [Ca^2+^]_cyt_. **(D)** Single molecule resolution images of K_Ca_2.2 channels in plasma membrane of irradiated (1.25 Gy) and non-irradiated Jurkat cells (scale bar = 1 µm). **(E)** Mean number of K_Ca_2.2 molecules in control cells (black symbols) and 48 h after irradiation (red symbols). Each circle represents an individual region of interest (ROI) of a single Jurkat cell. Box plot symbols have the same meaning as in Figure 2.

Current responses and corresponding current–voltage (I/V) relationships in mock and irradiated Jurkat cells are reported in Figures 7A,B. Hyperpolarizing voltage steps elicited only small currents in non-irradiated cells; voltages ≥−40 mV activated the outward rectifying Kv1.3 channel, which is prominently and constitutively expressed in Jurkat cells (36). During extended positive test pulses these channels were fully inactivated, resulting in a small background current I_b_ (Figure 7C inset). This small current includes, among others, the voltage independent small conductance K^+^ channel K_Ca_2.2. Subtraction of the latter from the peak current provides a measure for the Kv1.3 channel (I_Kv_).

To evaluate the effect of IR on the relative contribution of K_Ca_2.2 to the total current, we measured Jurkat cells under four different conditions: (i) mock-irradiated cells with low (≤100 nM) and (ii) high (1 µM) [Ca^2+^]_cyt_ as well as irradiated cells with (iii) low or (iv) high internal [Ca^2+^]_cyt_ (Figure 7D). Data given in Figure 7 indicate that I_b_/I_kv_ is not augmented by an elevation of [Ca^2+^]_cyt_. This situation is different in irradiated cells where a high [Ca^2+^]_cyt_ caused a significant increase in the relative conductance of I_b_. An example for the currents and the corresponding I/V relation from an irradiated cell measured with high [Ca^2+^]_cyt_ is shown in Figure 7. The increase in I_b_ is most apparent in the elevated instantaneous activating inward current. To test whether this additional conductance includes K_Ca_2.2 activity cells were treated with the scorpion toxin Tamapin (10 nM), a specific high affinity inhibitor (IC_50_ = 24 pM) of K_Ca_2.2 channels (37). Treatment revealed a marginal 3.3 ± 0.7% (*N* = 3) inhibition of I_b_ in mock-irradiated control cells while Tamapin resulted in a 23 ± 10% inhibition of the respective current in irradiated cells.

To further test the IR triggered upregulation of K_Ca_2.2 channels, their density in the plasma membrane was analyzed by high-resolution single molecule microscopy. The representative images in Figures 7D,E show that irradiation caused an elevated number of fluorescent signals in the plasma membrane of Jurkat cells after 48 h (Figure 7E). The mean number of fluorescent signals from immunostained K_Ca_2.2 molecules was 1.3 times higher in irradiated cells compared to controls.

## Discussion

The relationship between IR and the activation or suppression of the immune system is considered complex and multifactorial. It strictly depends on the dose applied as well as on the type and differentiation status of the immune cell type investigated (2, 38). X-irradiation with single doses ≥2 Gy used in clinical oncological practice generally triggers activating (pro-inflammatory) functions to mediate toxic and/or immune stimulatory effects of RT (1, 3). Application of low-dose radiotherapy with single doses <1 Gy on the other hand is reported to mediate anti-inflammatory effects in a multitude of benign disorders (39, 40).

During the last decades, multiple efforts have been made to uncover the molecular events following radiation exposure and subsequent irradiation-triggered pathways including induction of an inflammatory response (41, 42). We have recently reported that an increase in ROS following X-irradiation with doses ≥1 Gy results in both nuclear and cytoplasmic detection in malignant cells (11, 12). An increase in cytosolic ROS further triggers a Ca^2+^-mediated signal transduction cascade, which eventually activates Ca^2+^ sensitive K^+^ channels and causes membrane hyperpolarization (11, 12). Moreover, upon contact with antigen presenting cells, mitogens or IR, T-lymphocytes respond with a rise in [Ca^2+^]_cyt_ (43–45). This elicits a multitude of responses, including protein expression, altered phosphorylation patterns, induction of transcription factors (13–16) and an increase in cell diameter (46). In this study, we observed that IR causes also in Jurkat cells a Ca^2+^ signaling cascade, which was not an immediate consequence of irradiation but triggered only after a considerable delay. The same treatment furthermore enhanced expression of the IL-2 receptor (CD25), and cytokines IFNγ and IL-2 at least in Jurkat cells, elevated levels of integrin β1-mediated cell adhesion, augmentation in the conductance of the Ca^2+^ sensitive K_Ca_2.2 channel and a dose-dependent increase in cell diameter. Collectively, this indicates that IR presumably affects an immunological activation or modulation of these cells. In favor of the view that the increase in cell diameter is related to immune activation and Ca^2+^ dependent, we monitored a 50% reduction of the cell diameter increase upon treatment with the immunosuppressant cyclosporine A or by buffering changes in [Ca^2+^]_cyt_ by BAPTA-AM. Moreover, we recognized an IR-induced G2 cell cycle arrest that correlated to the increase in cell diameter by an increase in the size of the nucleus. Consequently, the IR induced increase in cell diameter can be dissected at least in two components, a Ca^2+^-mediated immune stimulation and a radiation-induced cell cycle arrest.

Adhesion of immune cells to the endothelium displays an initial step in inflammatory cascades and recruitment of T-lymphocytes from peripheral blood to tumor tissue sites (47). Here, we indicate that single doses of 1.25 Gy (48) increase Jurkat T-cell adhesion to Ea.hy926 ECs. The IR-induced increase in adhesion of Jurkat cells was significantly inhibited by addition of peptide comprising the three amino acids Arg–Gly–Asp (RGD peptide) indicating a predominant involvement of integrin β1 adhesion molecules (49) but less pronounced for PBL. This may be attributed to the heterogeneity of cell populations in PBL suspensions with different sets of adhesion molecule expression. By combined immunostaining and high-resolution single molecule microscopy resolving an increased expression, we further confirmed clustering of integrin β1 molecules on Jurkat cells to contribute to the adhesion process. Although not detailed in the present investigation, the underlying mechanism(s) seem to be multifactorial. They may include radiation-induced activation of a variety of transcription factors like the immune relevant nuclear factor kappa B (50). The latter was recently reported to directly bind the integrin β1 promotor region in response to IR resulting in an upregulation of the subunit and modulation of invasiveness and radiation resistance (51).

The IR triggered altered adhesion properties may have different consequences: inflammatory IR responses can favor malignant cell invasion, providing a favorable environment for tumor promotion and metastasis (52–54) or secondary malignancies (55). By this, IR may alter cell phenotypes, which in turn contribute, directly or indirectly, to carcinogenesis. It may also affect the activity or abundance of tissue proteases, growth factors, cytokines and adhesion molecules, which are involved in tissue remodeling (56).

This study mainly focused on the established Jurkat model for analyzing immunological effects of IR but exemplary experiments were also performed on PBL from healthy blood donors indicating differences in CD25 surface detection, cytokine IFNγ and IL-2 expression, and integrin-mediated adhesion to ECs. There is compelling evidence that subpopulations of T cells may display differential radiation sensitivities. While T helper lymphocytes and cytotoxic T cells are characterized by a radiation sensitive phenotype, regulatory T cells, appear to be more radioresistant (38). Notably, by comparing the effects of IR on gene expression in CD4+ T lymphocytes and in Jurkat cells, Mori et al. reported on a predominat upregulation of p53 target genes in naïve CD4+ positive cells. By contrast, Jurkat leukemic cells with a non-functional p53 gene are characterized by alterations in a more limited set of genes belonging to the Rho GTPase and cytokine signaling pathways (57). Accordingly, one may assume that activation of CD25 expression and cytokine response in Jurkat versus PBL may arise from a differential (p53 dependent) gene activation.

More recently, however, it has further become evident that IR not only induces inflammatory reactions and unwanted, temporary immune suppression like leukopenia but is also capable of triggering specific anti-tumor immune responses. This occurs especially when IR is applied in multimodal settings in combination with checkpoint cytotoxic T-lymphocyte-associated protein 4 and programmed death PD-1 and its ligand PD-L1 inhibitors (1, 8). In line with that, distinct tumor infiltrating immune cells, most relevant cytotoxic CD8+ T-cells, predict the response to radio(chemo)therapy in a multitude of tumor entities and display an essential prerequisite for successful radio-immune therapeutic strategies (4–6).

In summary, our findings indicate that IR in a clinically relevant dose may foster immune activation and functional properties of T-lymphocytes that may have implications for both toxic and cancer inducing effects of radiotherapy but also increases tumor response to combined RT and novel immune therapies in cancer patients and patients with non-malignant disorders.

## Author Contributions

PV, SF, FW, LB, SH, and DT performed experiments and analyzed data. PV, TM, CF, SH, FR, AM, and GT designed experiments and analyzed/interpreted data. PV, CF, AM, FR, and GT wrote the paper. All authors were critically revising the work and approved the final content.

## Conflict of Interest Statement

The authors declare that the research was conducted in the absence of any commercial or financial relationships that could be construed as a potential conflict of interest.

## Supplementary Material

Figure S1 Ionizing irradiation stimulates adhesion of peripheral blood lymphocytes (PBL). Relative adhesion rates of PBL to endothelial cells. Cells were incubated at 4°C (open circles) or 37°C (closed circles) without (−) or with (+) TNF-α (20 ng/ml). Cell were further treated with CD3/CD28/CD2 T-cell activator (25 µl/ml), irradiated with 1.25 Gy X-ray in absence or presence of 10 µM RGD peptide in incubation buffer. All data were normalized to value measured for untreated cells at 4°C. Mean values ± SD (*n* = 5; *N* = 3). Student’s *t*-test compared activator-treated and irradiated cells with non-irradiated controls with TNF-α, 37°C and irradiated cells with and without RGP peptide; **P* < 0.05, ***P* < 0.01.

Figure S2 Irradiation stimulates immune activation in Jurkat cells. Western immune blots **(A)** and quantification **(B)** of Jurkat cells 48 h after irradiation with a dose of 1.25 and 2 Gy using an anti-CD25 antibody. Mock-irradiated cells served as a control (*n* = 3). Immuno-fluorescent detection (scale bar = 25 µm) **(C)** and quantification **(D)** of interleukin-2 (IL-2) in control and Jurkat cells 48 h after irradiation with 2 Gy. Mean fluorescent intensity ± SD in region of interests (ROIs) in the cytoplasm of control cells and irradiated cells (*N* = 2; *n* ≥ 10 cells). Immuno-fluorescent detection (scale bar = 25 µm) 10 **(E)** and quantification **(F)** of interferon-γ (IFNγ) in control and Jurkat cells 48 h after irradiation with X-ray doses between 0.5 and 5 Gy or treatment with 25 µl/ml CD3/CD28/CD2 T-cell activator. Mean fluorescent intensity ± SE in ROIs of control cells and irradiated cells (*N* = 3; *n* ≥ 70 cells). Data in **(F)** were fitted by Eq. 1 yielding a D50 value of 1 Gy and a maximum increase in fluorescence of 2.
