# Supplementary figures and images for: Ionizing Radiation Induces Morphological Changes and Immunological Modulation of Jurkat Cells
Source: Front Immunol. 2018 Apr 30;9:922. doi: 10.3389/fimmu.2018.00922 (PMC5936756; doi:10.3389/fimmu.2018.00922)

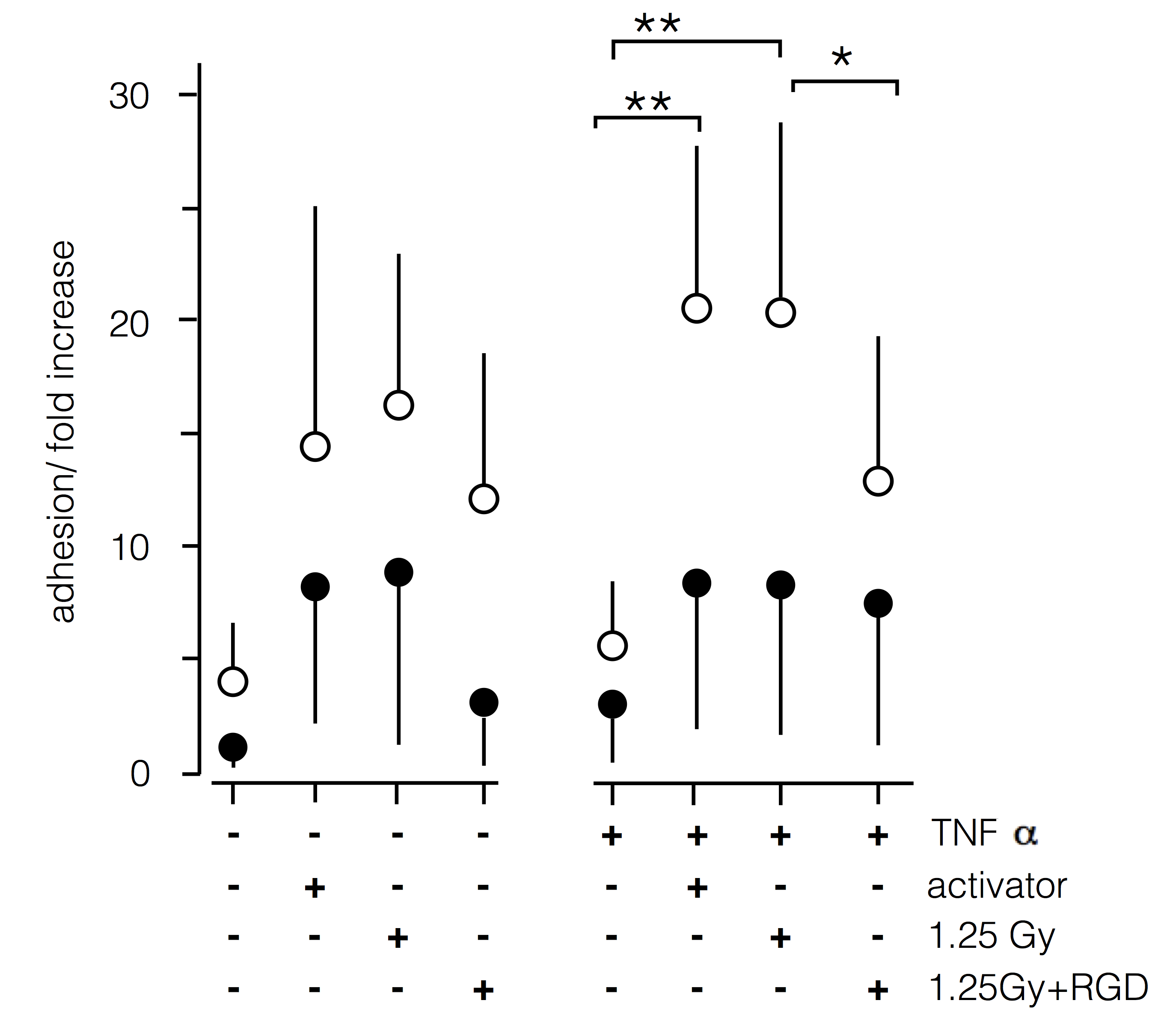

Supplement: Figure S1 — Ionizing irradiation stimulates adhesion of peripheral blood lymphocytes (PBL). Relative adhesion rates of PBL to endothelial cells. Cells were incubated at 4°C (open circles) or 37°C (closed circles) without (−) or with (+) TNF-α (20 ng/ml). Cell were further treated with CD3/CD28/CD2 T-cell activator (25 µl/ml), irradiated with 1.25 Gy X-ray in absence or presence of 10 µM RGD peptide in incubation buffer. All data were normalized to value measured for untreated cells at 4°C. Mean values ± SD (n = 5; N = 3). Student’s t-test compared activator-treated and irradiated cells with non-irradiated controls with TNF-α, 37°C and irradiated cells with and without RGP peptide; *P < 0.05, **P < 0.01. [file image_1.tiff]

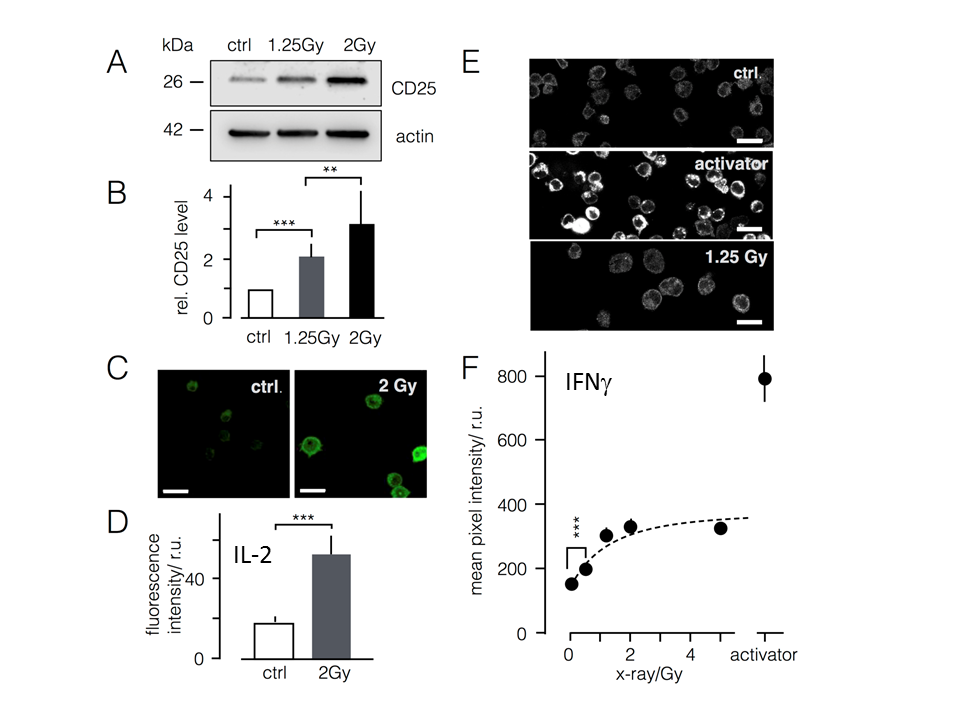

Supplement: Figure S2 — Irradiation stimulates immune activation in Jurkat cells. Western immune blots (A) and quantification (B) of Jurkat cells 48 h after irradiation with a dose of 1.25 and 2 Gy using an anti-CD25 antibody. Mock-irradiated cells served as a control (n = 3). Immuno-fluorescent detection (scale bar = 25 µm) (C) and quantification (D) of interleukin-2 (IL-2) in control and Jurkat cells 48 h after irradiation with 2 Gy. Mean fluorescent intensity ± SD in region of interests (ROIs) in the cytoplasm of control cells and irradiated cells (N = 2; n ≥ 10 cells). Immuno-fluorescent detection (scale bar = 25 µm) 10 (E) and quantification (F) of interferon-γ (IFNγ) in control and Jurkat cells 48 h after irradiation with X-ray doses between 0.5 and 5 Gy or treatment with 25 µl/ml CD3/CD28/CD2 T-cell activator. Mean fluorescent intensity ± SE in ROIs of control cells and irradiated cells (N = 3; n ≥ 70 cells). Data in (F) were fitted by Eq. 1 yielding a D50 value of 1 Gy and a maximum increase in fluorescence of 2. [file image_2.tif]
